# Pemetrexed Induced Thymidylate Synthase Inhibition in Non-Small Cell Lung Cancer Patients: A Pilot Study with 3′-Deoxy-3′-[^18^F]fluorothymidine Positron Emission Tomography

**DOI:** 10.1371/journal.pone.0063705

**Published:** 2013-05-24

**Authors:** Virginie Frings, Astrid A. M. van der Veldt, Ronald Boellaard, Gerarda J. M. Herder, Elisa Giovannetti, Richard Honeywell, Godefridus J. Peters, Erik Thunnissen, Otto S. Hoekstra, Egbert F. Smit

**Affiliations:** 1 Department of Radiology and Nuclear Medicine, VU University Medical Center, Amsterdam, The Netherlands; 2 Department of Pulmonary Diseases, St. Antonius Hospital, Nieuwegein, The Netherlands; 3 Department of Medical Oncology, VU University Medical Center, Amsterdam, The Netherlands; 4 Department of Pathology, VU University Medical Center, Amsterdam, The Netherlands; 5 Department of Pulmonary Diseases, VU University Medical Center, Amsterdam, The Netherlands; NIH, United States of America

## Abstract

**Objectives:**

Pemetrexed is a thymidylate synthase (TS) inhibitor and is effective in non-small cell lung cancer (NSCLC). 3′-deoxy-3′-[18F]fluorothymidine (^18^F-FLT), a proliferation marker, could potentially identify tumor specific TS-inhibition. The aim of this study was to investigate the effect of pemetrexed-induced TS-inhibition on ^18^F-FLT uptake 4 hours after pemetrexed administration in metastatic NSCLC patients.

**Methods:**

Fourteen NSCLC patients underwent dynamic ^18^F-FLT positron emission tomography (PET) scans at baseline and 4 hours after the first dose of pemetrexed. Volumes of interest were defined with a 41%, 50% and 70% threshold of the maximum pixel. Kinetic analysis and simplified measures were performed. At one, two, four and six hours after pemetrexed, plasma deoxyuridine was measured as systemic indicator of TS-inhibition. Tumor response measured with response evaluation criteria in solid tumors (RECIST), time to progression (TTP) and overall survival (OS) were determined.

**Results:**

Eleven patients had evaluable ^18^F-FLT PET scans at baseline and 4 hours after pemetrexed. Two patients had increased ^18^F-FLT uptake of 35% and 31% after pemetrexed, whereas two other patients had decreased uptake of 31%. In the remaining seven patients ^18^F-FLT uptake did not change beyond test-retest borders. In all patients deoxyuridine levels raised after administration of pemetrexed, implicating pemetrexed-induced TS-inhibition. ^18^F-FLT uptake in bone marrow was significantly increased 4 hours after pemetrexed administration. Six weeks after the start of treatment 5 patients had partial response, 4 stable disease and 2 progressive disease. Median TTP was 4.2 months (range 3.0–7.4 months); median OS was 13.0 months (range 5.1–30.8 months). Changes in ^18^F-FLT uptake were not predictive for tumor response, TTP or OS.

**Conclusions:**

Measuring TS-inhibition in a clinical setting 4 hours after pemetrexed revealed a non-systematic change in ^18^F-FLT uptake within the tumor. No significant association with tumor response, TTP or OS was observed.

## Introduction

Non-small cell lung cancer (NSCLC) often presents in an advanced stage. Unfortunately, treatment options are limited at this stage, including chemotherapy with or without radiotherapy [1] and targeted therapies [2]. Therefore, despite new drugs and personalized therapy, treatment of metastatic NSCLC remains challenging.

Pemetrexed, an anticancer drug with clinical efficacy in non-squamous NSCLC, inhibits thymidylate synthase (TS) [3], dihydrofolate reductase (DHFR), and glycinamide ribonucleotide formyltransferase (GARFT) [4]. It is used as first line treatment in combination with cisplatin or carboplatin and as monotherapy in second line treatment in metastatic NSCLC. In the literature, response rates of pemetrexed vary between 10–30% [5]. Level of TS expression showed an inverse correlation with pemetrexed sensitivity [6]. Pemetrexed has several side-effects such as nausea, anemia, bone marrow depression, stomatitis, pharyngitis and rash [7], [8], which can be severe. Toxicities could be reduced in non-responding patients if effectiveness would be predictable, preferably in an early stage, e.g. from positron emission tomography (PET) measurements.

**Figure 1:**
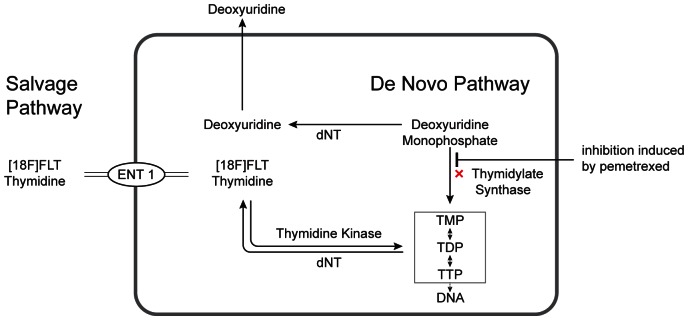
Cellular pathway of thymidine with the interaction of pemetrexed indicated. ^18^F-FLT is not incorporated into DNA, as shown by the dotted arrow. Abbreviations: ENT, Equilibrative Nucleoside Transporter; TMP, Thymidine Monophosphate; TDP, Thymidine Diphosphate; TTP, Thymidine Triphospate; dNT, deoxyribonucleotidase.

3′-deoxy-3′-[^18^F]fluorothymidine (^18^F-FLT) PET could function as non-invasive biomarker of TS-inhibition effectuated by pemetrexed. TS is a key enzyme for the synthesis of deoxyribonucleic acid (DNA) and as such a target for anticancer drugs. Figure 1 visualizes the cellular pathway of thymidine, which consists of the de novo and the salvage pathway. TS is the essential enzyme in the de novo pathway of thymidine nucleotides. When the de novo pathway is down regulated by a TS inhibitor (pemetrexed), DNA synthesis will depend on the salvage pathway, which will be up regulated, facilitated by redistribution of the equilibrative nucleoside transporter (ENT) to the cell membrane [9]. Figure 1 indicates the interaction of pemetrexed, which is TS inhibition. ^18^F-FLT follows the salvage pathway of endogenous thymidine, which also provides thymidine nucleotides. However, unlike endogenous thymidine, ^18^F-FLT is trapped in the cytosol and is not incorporated into DNA. The uptake of ^18^F-FLT will increase as a result of the up regulation of the salvage pathway, when TS is effectively inhibited. In addition, inhibition of thymidylate synthase will lead to accumulation of deoxyuridine monophosphate which will be broken down to deoxyuridine and released to the extracellular compartment and plasma. An increase of plasma deoxyuridine after TS inhibition treatment may be considered as a systemic surrogate marker of TS-inhibition. ^18^F-FLT PET could monitor tumor specific changes of ^18^F-FLT uptake after TS-inhibiting treatment [9].

The first clinical study of imaging drug-induced TS-inhibition showed an increased [11C]thymidine uptake one hour after nolatrexed (TS-inhibitor) administration in gastrointestinal cancer patients [10]. A mouse model (fibrosarcoma) showed that ^18^F-FLT PET allows early measurement of TS-inhibition caused by 5–fluorouracil, with a 1.8 fold increase of ^18^F-FLT uptake 1–2 hours after treatment [11]. This increase coincided with a twofold increase in deoxyuridine accumulation in plasma. Hence, ^18^F-FLT PET appears suited for non-invasive assessment of TS-inhibition in tumors.

Since ^18^F-FLT signal harbours combined information of proliferation and TS-inhibition, appropriate timing of PET after administration of pemetrexed is important: if applied too late, the proliferation signal may dominate (a decline in case of response), whereas the actual TS-inhibition may be most prominent within the first 24 hours. Preclinical ^18^F-FLT PET data was derived two hours after intervention. Earlier data [12] suggested that this time-schedule could be extended in patients since the deoxyuridine accumulation is present up to six hours, therefore, a PET scan after 4 hours was chosen as the optimal time interval.

The aim of the present study was to investigate the effect of pemetrexed-induced TS-inhibition on ^18^F-FLT uptake 4 hours after pemetrexed administration in patients with metastatic NSCLC.

## Materials and Methods

### Patients

Fourteen patients with histological confirmed NSCLC adenocarcinoma were included prospectively. Patients were recruited from the VU University Medical Center in Amsterdam, The Netherlands. Patients had to be ≥18 years old, scheduled for treatment with pemetrexed and had a tumor of ≥3 cm in diameter within the chest. Prior to study enrolment, all patients signed a protocol-specific informed consent. Patients were staged according to the 7^th^ TNM classification system developed by International Association for the Study of Lung Cancer [13] and the Eastern Cooperative Oncology Group (ECOG) performance status [14] were determined. Patients underwent ^18^F-FLT PET scanning within one week before the start of treatment with pemetrexed and 4 hours after the first therapeutic pemetrexed dose, which consisted of 500 mg·m^−2^. ^18^F-FLT PET scans and deoxyuridine measurements were performed after pemetrexed as single agent. Combination therapy with cisplatin (75 mg·m^−2^) or carboplatin (AUC 5) was given one day later to avoid interference with ^18^F-FLT uptake. Treatment schedule was according to standard practice, which is every 3 weeks. The study was approved by the medical ethics review committee of the VU University Medical Center.

### PET Imaging

PET scans were performed using an ECAT EXACT HR+ scanner (Siemens/CTI), which consists of 32 rings of bismuth germanate oxyorthosilicate (BGO) detectors [15]. This scanner enables the acquisition of 63 planes of data over a 15.5 cm axial field of view. Two venous cannula were inserted: one was used for tracer injection and the other for blood sampling.

First, a 10 minute transmission scan was performed using three retractable rotating line sources. These data were used to correct the subsequent emission scans for photon attenuation. Following the transmission scan, a target dose of 250 MBq ^18^F-FLT was injected intravenously, 30 seconds after starting a dynamic emission scan in 3D setting with a total scan time of 60.5 minutes and with the following frame lengths: 6×5 s, 6×10 s, 3×20 s, 5×30 s, 5×60 s, 8×150 s, and 6×300 s. All emission scans were reconstructed using filtered back projection (FBP) with a 0.5 Hanning filter, resulting in a transaxial spatial resolution of ∼7 mm in the center of the field of view.

Venous samples were drawn at 5, 10, 20, 30, 40 and 60 minutes after ^18^F-FLT injection. Whole blood and plasma activity was measured immediately, as were plasma metabolite fractions.

### PET Data Analysis

The primary lesion was identified in all patients. Volume of interest (VOI) were defined using a semiautomatic threshold technique on the summed last 3 frames, which were reconstructed using ordered subset expectation maximisation reconstruction with 2 iterations and 16 subsets and 5 mm full width at half maximum (FWHM) Gaussian smoothing. The VOI thresholds 41% and 70% of the maximum pixel corrected for background, and 50% corrected and uncorrected for background were analysed, together with the maximal pixel in the VOI. Tumor VOIs were transferred to FBP reconstructed dynamic ^18^F-FLT images to generate time-activity curves (TAC).

An image-derived input function (IDIF) was obtained by manually drawing 2-dimensional regions of interest over the aortic arch, aorta ascendens and aorta descendens on FBP reconstructed images, these were then applied to all frames to generate an IDIF. The IDIFs were corrected for plasma-to-blood ratio and parent fractions to obtain metabolite corrected plasma input functions.

For each VOI, several (semi)quantitative methods were calculated using Matlab (Natick, MA). Standardized uptake values (SUV) were calculated for the interval 40–60 minutes and 50–60 minutes, normalised to body weight, lean body mass and body surface area. Total lesion proliferation (TLP) was calculated as metabolic volume *SUV. Full kinetic analysis with non linear regression (NLR), using irreversible and reversible two tissue compartment models with 3 and 4 parameters respectively, were included, together with blood volume fraction. Patlak analysis [16], assuming an irreversible model and resulting in net uptake rate K_i_, with the time intervals 10–60 minutes, 10–45 minutes, 10–30 minutes, 20–60 minutes and 30–60 minutes were assessed. Volume of distribution was calculated as K_1_/k_2_*(1+k_3_/k_4_), with the parameters derived from NLR with 4 kinetic rate constants.

In addition, mean SUV corrected for bodyweight were calculated for muscle, liver, bone marrow and lung tissue, as non-tumor reference tissue. These VOI were placed manually over the designated tissue in a standardized manner (muscle 3×3 voxels in 5 planes, liver 7×7 voxels in 5 planes, bone marrow 2×2 voxels in 5 planes, lung 3×3 voxels in 5 planes).

### Deoxyuridine Measurements in Plasma

Plasma samples for deoxyuridine were taken at least one week and one minute before the first dose of pemetrexed, functioning as double baseline measurements. Subsequently, samples were taken one, two, four and six hours after pemetrexed to determine the deoxyuridine plasma concentration over time. Deoxyuridine (ng/ml) was measured with a validated liquid chromatography with tandem mass spectrometry detection (LC-MS/MS) assay as described earlier [17], [18].

### Immunohistochemistry

The procedure for immunohistochemistry (IHC) for TS expression was slightly modified from Van Triest [19]. Thymilydate synthase mouse monoclonal Mouse Clone TS106, DAKO, 1/100, for 1 hr at room temperature. Detection system PowerVision was used. TS expression was scored quantitatively with the H-score in nucleus and cytoplasma. H-score was scored as ∑(*I* × PC), where *I* represents the staining intensity and PC the percentage of cells that stain at each intensity. The median H-score of the included patients was used as cut-off value for low and high TS expression classification.

### TS and Methylenetetrahydrofolate Reductase (MTHFR) Polymorphism

TS and MTHFR polymorphism are prognostic factors in NSCLC [20]. Isolated leucocytes from the first blood sample were used to determine TS and MTHFR polymorphism with real time polymerase chain reaction [21]. We defined 2R2R, 2R3R and 3R3R polymorphism for TS and 677C, 677T and C677T for MTHFR.

### Clinical Outcome

Tumor response was evaluated after six weeks on computed tomography (CT) according to response evaluation criteria in solid tumors (RECIST) [22]. Furthermore, time to progression (TTP) and overall survival (OS) were defined as the start of treatment with pemetrexed until the first observation of tumor progression and the day of death, respectively.

### Statistical Analysis

The primary outcome was the absolute and relative difference of ^18^F-FLT uptake measured with SUV and full kinetic analysis 4 hours after the first dose of pemetrexed beyond earlier established test-retest boundaries [23]. The differences in ^18^F-FLT uptake were correlated with clinical outcome measured as tumor response, TTP and OS. Variables were tested for normality to decide for parametric or nonparametric statistics and a p-value <0.05 was considered significant. Statistical analysis consisted of paired t-test, Wilcoxon signed-rank test and Kruskal-Wallis test. The median value for TTP and OS was calculated with Kaplan Meier. All statistical analyses were performed using SPSS 15.0.

## Results

### Patients

Fourteen NSCLC patients with stage IV disease were included. Performance score was 0–1 for all patients, median age was 59 and six patients were male. Three patients were treatment naïve, eleven patients had 1^st^ line treatment and three patients had 2^nd^ line chemotherapy before treatment with pemetrexed. None of the patients had radiotherapy targeted on the index lesions. After study enrolment seven out of fourteen patients received pemetrexed in combination with carboplatin, six in combination with cisplatin and one patient received pemetrexed monotherapy.

### PET Scans

From the fourteen included patients, eleven patients were evaluable for deriving changes in ^18^F-FLT (see Table 1 for descriptive statistics). Three patients were not evaluable due to: (1) technical errors with the PET scanner, (2) insufficient tracer production, and (3) non-evaluable kinetic parameters for the second ^18^F-FLT PET scan. ^18^F-FLT dosage ranged from 226–278 MBq. From the tested VOI, 50% corrected for background was most suitable, because it was feasible for all but one lesion and has been shown to be the VOI of preference as described in previous work [24]. In addition, study conclusions were not affected by VOI method. Therefore, further results shown are based on the VOI 50% with background correction and normalised to body weight.

**Table 1:** Patient demographics and characteristics.

Characteristics		Total no. of patients n = 14 (%)	PET evaluable patients n = 11 (%)
Sex	Male	6 (43)	3 (27)
	Female	8 (47)	8 (73)
Age (years)	Median	59	61
	Range	35–77	35–77
TS polymorphism	2R2R	5 (35.7)	4 (36.4)
	3R3R	3 (21.4)	2 (18.2)
	2R3R	4 (28.6)	4 (36.4)
	Missing data	2 (14.3)	1 (9.1)
MTHFR polymorphism	677C	6 (42.9)	5 (45.5)
	677T	3 (21.4)	3 (27.3)
	C677T	3 (21.4)	2 (18.2)
	Missing data	2 (14.3)	1 (9.1)
Chemotherapy	Pemetrexed	1 (7.1)	1 (9.1)
	Pemetrexed/carboplatin	7 (50)	5 (45.5)
	Pemetrexed/cisplatin	6 (42.9)	5 (45.5)
RECIST	Complete response	0 (0)	0 (0)
	Partial response	5 (35.7)	5 (45.5)
	Stable disease	7 (50.0)	4 (36.4)
	Progressive disease	2 (14.3)	2 (18.2)
TTP (months)	Median	4.3	4.2
	Range	3.0–10.4	3.0–7.4
Overall Survival (months)	Median	9.7	13.0
	Range	5.1–32.7	5.1–30.8

Abbreviations: TS, Thymidylate Synthase; MTHFR, methylenetetrahydrofolate reductase; RECIST, Response Evaluation Criteria in Solid Tumors; TTP, Time To Progession.

Kinetic analysis was performed to validate SUV against full quantitative measures (e.g. K_i_ obtained using non linear regression and Patlak analysis). Table 2 shows the median kinetic parameters for the reversible and irreversible NLR model, Patlak analysis and SUV for the paired scans. The reversible two tissue compartment model with 4 parameters was the model of choice for NLR as indicated by the Akaike and Schwarz criteria [25] in 7 out of 11 (64%) for the baseline scans. For the scans 4 hours after pemetrexed administration Akaike indicated the best fit with the reversible two tissue compartment model in 8 out of 11 (73%) and Schwarz in 7 out of 11 (64%) scans. NLR K_i_ results shown in this paper are therefore based on the reversible two tissue compartment model with 4 kinetic rate constants.

**Table 2:** Results of standardized uptake value, non linear regression and Patlak K_i_ analysis per scan.

	Baseline Median (range)	4 hours after pemetrexed administration Median (range)
SUV	3.7 (2.2–8.0)	4.0 (1.7–8.9)
NLR 3k K_1_	0.201 (0.010–0.353)	0.171 (0.095–13.30)
NLR 3k k_2_	0.103 (0.009–0.647)	0.098 (0.049–0.175)
NLR 3k k_3_	0.026 (0.011–0.161)	0.022 (0.012–0.041)
NLR 3k K_i_	0.032 (0.010–0.079)	0.031 (0.010–0.060)
NLR 4k K_1_	0.256 (0.019–0.443)	0.220 (0.123–11.40)
NLR 4k k_2_	0.267 (0.042–1.230)	0.169 (0.045–0.324)
NLR 4k k_3_	0.081 (0.011–1.350)	0.038 (0.011–0.161)
NLR 4k k_4_	0.020 (0.000–0.032)	0.006 (0.000–0.032)
NLR 4k K_i_	0.048 (0.010–0.136)	0.035 (0.023–0.076)
Patlak K_i_	0.026 (0.009–0.061)	0.028 (0.010–0.52)

**Figure 2:**
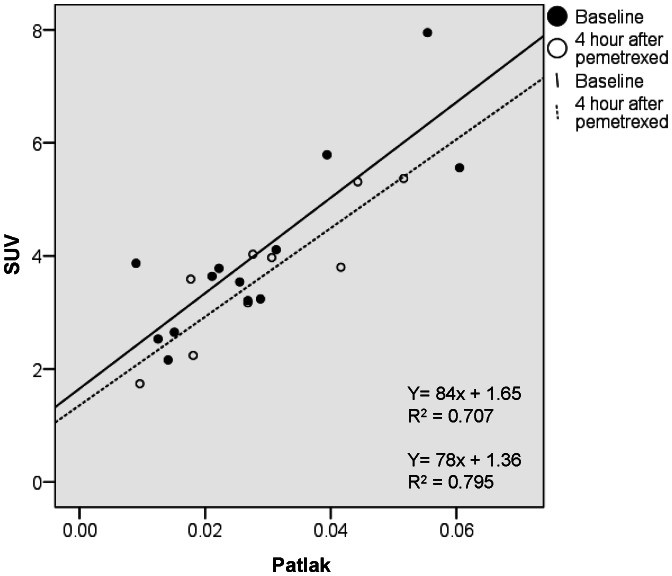
Correlation between SUV and Patlak at baseline and 4 hours after pemetrexed administration.

The median volume of distribution, derived from NLR 4k was 4.1 ml·cm^−3^ and 6.7 ml·cm^−3^ for baseline and 4 hours after pemetrexed administration respectively (Wilcoxon signed-rank test p = 0.60). The correlation between SUV and K_i_ derived from Patlak with different time intervals was moderate, with R-squares varying from 0.7–0.8. Figure 2 shows the correlation between SUV and Patlak K_i_ 10–60 minutes before and after treatment with pemetrexed. No statistical difference in the correlation between SUV and Patlak was found before and after the start of treatment with pemetrexed. Therefore SUV could be used to measure ^18^F-FLT uptake differences in this setting.

The median plasma to blood ratio and parent fraction for baseline and 4 hours after pemetrexed administration are shown in table 3. The median plasma to blood ratio ranged from 1.075–1.191. Twenty percent of ^18^F-FLT is glucuronidated in the liver at sixty minutes after ^18^F-FLT administration. There was no significant difference in plasma to blood ratio or parent fraction between the two scans.

**Table 3:** Plasma to blood ratio and parent fraction per scan.

	Baseline PET		PET 4 hours after pemetrexed administration	
Time (min)	Plasma to blood ratio Median (range)	Parent fraction Median (range)	Plasma to blood ratio Median (range)	Parent fraction Median (range)
5	1.075 (1.049–1.103)	98.49 (97.01–99.42)	1.075 (1.050–1.095)	98.36 (96.67–99.56)
10	1.085 (1.057–1.154)	95.75 (90.38–98.11)	1.093 (1.031–1.128)	95.64 (91.33–97.07)
20	1.116 (1.043–1.159)	90.62 (84.88–95.49)	1.119 (1.048–1.154)	90.03 (73.85–94.20)
30	1.148 (1.073–1.204)	86.70 (78.51–91.12)	1.142 (1.076–1.174)	83.21 (76.76–88.41)
40	1.166 (1.067–1.224)	83.28 (77.95–88.26)	1.167 (1.091–1.216)	79.91 (69.01–85.69)
60	1.185 (1.097–1.209)	80.55 (75.36–85.21)	1.191 (1.110–1.224)	79.15 (67.26–83.70)

**Figure 3:**
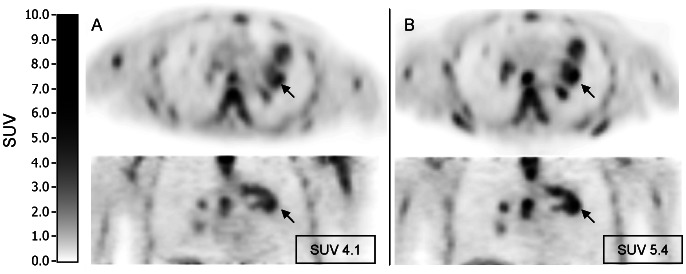
[18F]FLT PET scan. Example of ^18^F-FLT uptake (A) before and (B) 4 hours after pemetrexed administration, showing an increase of ^18^F-FLT uptake in the primary tumor (arrow) of 32% after pemetrexed administration.

**Figure 4:**
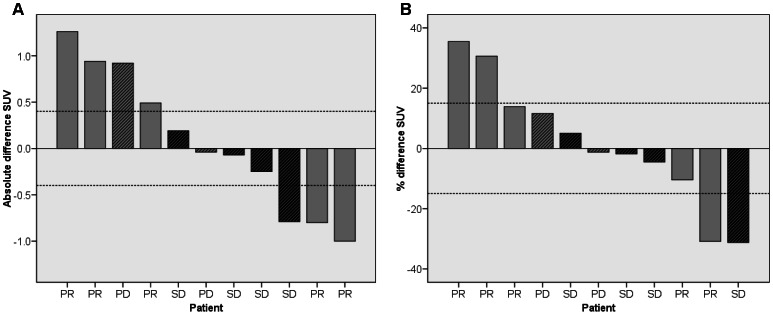
Change of ^18^F-FLT uptake. Change of ^18^F-FLT uptake in SUV 40–60 min normalised to bodyweight (A) absolute difference, (B) percentage difference.

**Figure 5:**
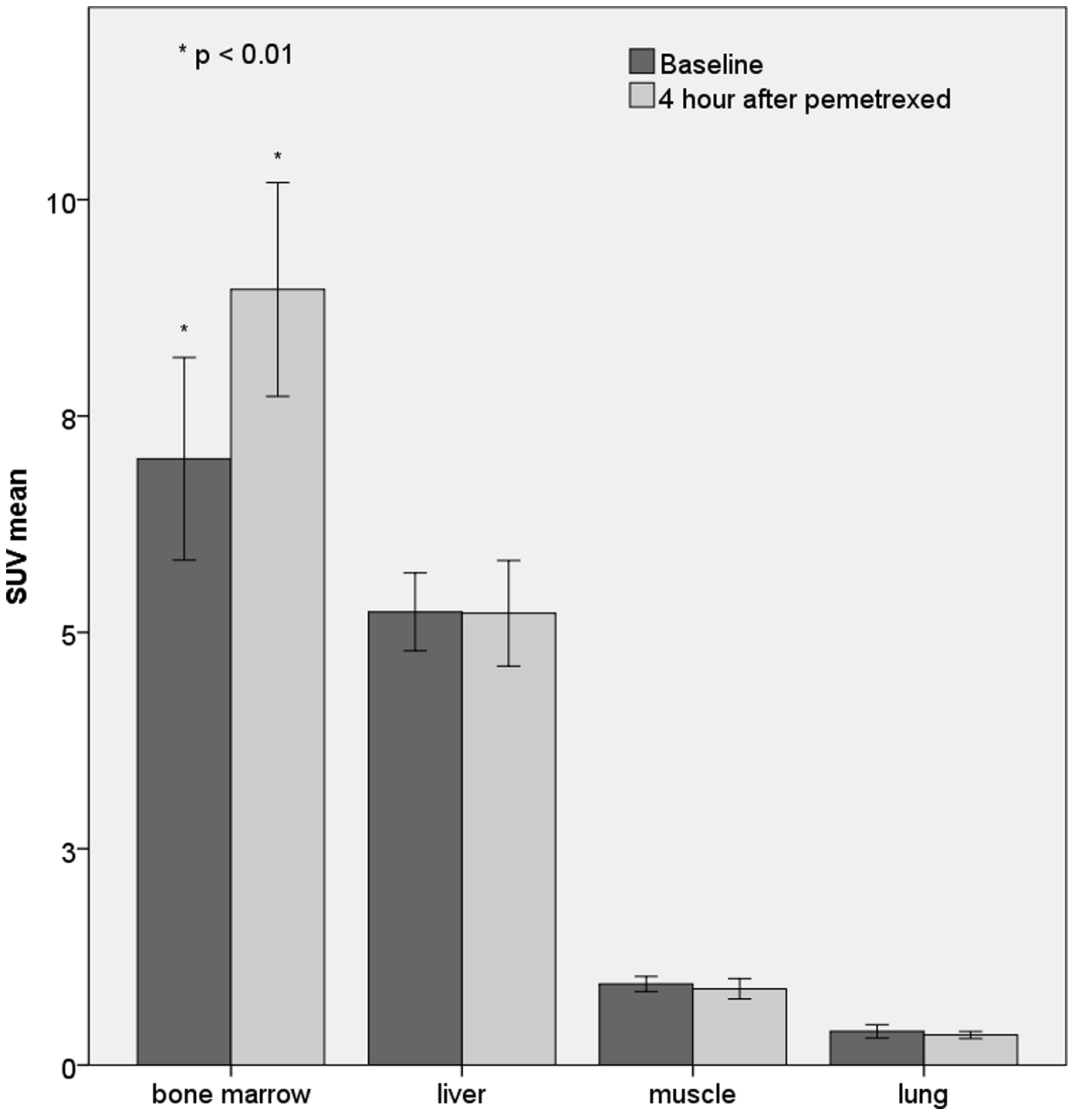
Mean SUV in muscle, liver, bone marrow and lung. Mean SUV normalised to bodyweight for muscle, liver, bone marrow and lung at baseline and 4 hours after pemetrexed.

In two patients, ^18^F-FLT tumor uptake significantly increased (31 and 35%) 4 hours after therapy compared with baseline (beyond test-retest borders of 15% [23]), while two other patients showed a significant decrease (31%). Figure 3 shows an example of a ^18^F-FLT PET scan of a patient with increased ^18^F-FLT uptake after pemetrexed administration. In the remaining seven patients, the change in ^18^F-FLT uptake was within the test-retest variability (see Figure 4). In Table 4 the results for the two ^18^F-FLT PET scans and the clinical outcome are listed per patient. The median SUV at baseline and 4 hours after pemetrexed administration were 3.89 and 3.78 (Wilcoxon signed-rank test p = 0.79). Median volume of the VOI at baseline was 11.6 ml, and 10.5 ml 4 hours after pemetrexed administration (p = 0.93). Median TLP was 38.5 and 37.8 for baseline and 4 hours after pemetrexed administration respectively (p = 0.42). Mean SUV in muscle, liver, bone marrow and lung are shown in Figure 5. Bone marrow exclusively showed a significant increase of ^18^F-FLT uptake of 33%, 4 hours after pemetrexed administration (p<0.01).

**Table 4:** Study results per patient.

	Baseline PET		PET 4 hours after pemetrexed administration		Abs.diff. PET		%diff. PET		Clinical Outcome		
Pt	SUV	NLR 4k K_i_ (ml·min^−1^·ml^−1^)	SUV	NLR 4k K_i_ (ml·min^−1^·ml^−1^)	SUV	NLR 4k K_i_ (ml·min^−1^·ml^−1^)	SUV (%)	NLR 4k K_i_ (%)	RECIST	TTP (months)	OS (months)
1	3.78	0.060	3.97	0.073	0.19	0.013	5.03	21.83	SD	4.3	7.7
2	2.16	0.022	–	–	–	–	–	–	SD	5.2	7.8
3	3.24	0.041	2.24	0.036	−1	−0.005	−30.86	−12.08	PR	5.6	19.2
4	5.56	0.133	5.31	0.048	−0.25	−0.085	−4.50	−64.06	SD	4.2	9.7
5	2.65	0.020	3.59	0.023	0.94	0.002	35.47	13.57	PR	7.4	27.4
6	2.53	0.023	1.74	0.031	−0.79	0.008	−31.23	34.96	SD	4.2	5.1
7	3.87	0.010	3.80	0.034	−0.07	0.024	−1.81	240.08	SD	4.0	32.7
8	3.64	0.019	–	–	–	–	–	–	SD	10.4	–
9	7.66	0.033	6.86	0.031	−0.8	0.001	−10.44	3.99	PR	6.5	13.0
10	7.95	0.136	8.87	–	0.92	–	11.57	–	PD	3.0	30.8
11	4.11	0.083	5.37	0.076	1.26	−0.007	30.66	−8.70	PR	3.7	7.3
12	3.54	0.063	4.03	0.067	0.49	0.004	13.84	6.34	PR	–	–
13	5.79	0.074	–	–	–	–	–	–	SD	5.8	5.9
14	3.21	0.055	3.17	0.032	−0.04	−0.023	−1.25	−42.23	PD	3.1	9.4

Abbreviations: SUV, Standardized Uptake Value; RECIST, Response Evaluation Criteria in Solid Tumors; SD, Stable Disease; PR, Partial Response; PD, Progressive Disease; TTP, Time To Progession in days; OS, Overall Survival in days.

### Clinical Outcome

**Figure 6:**
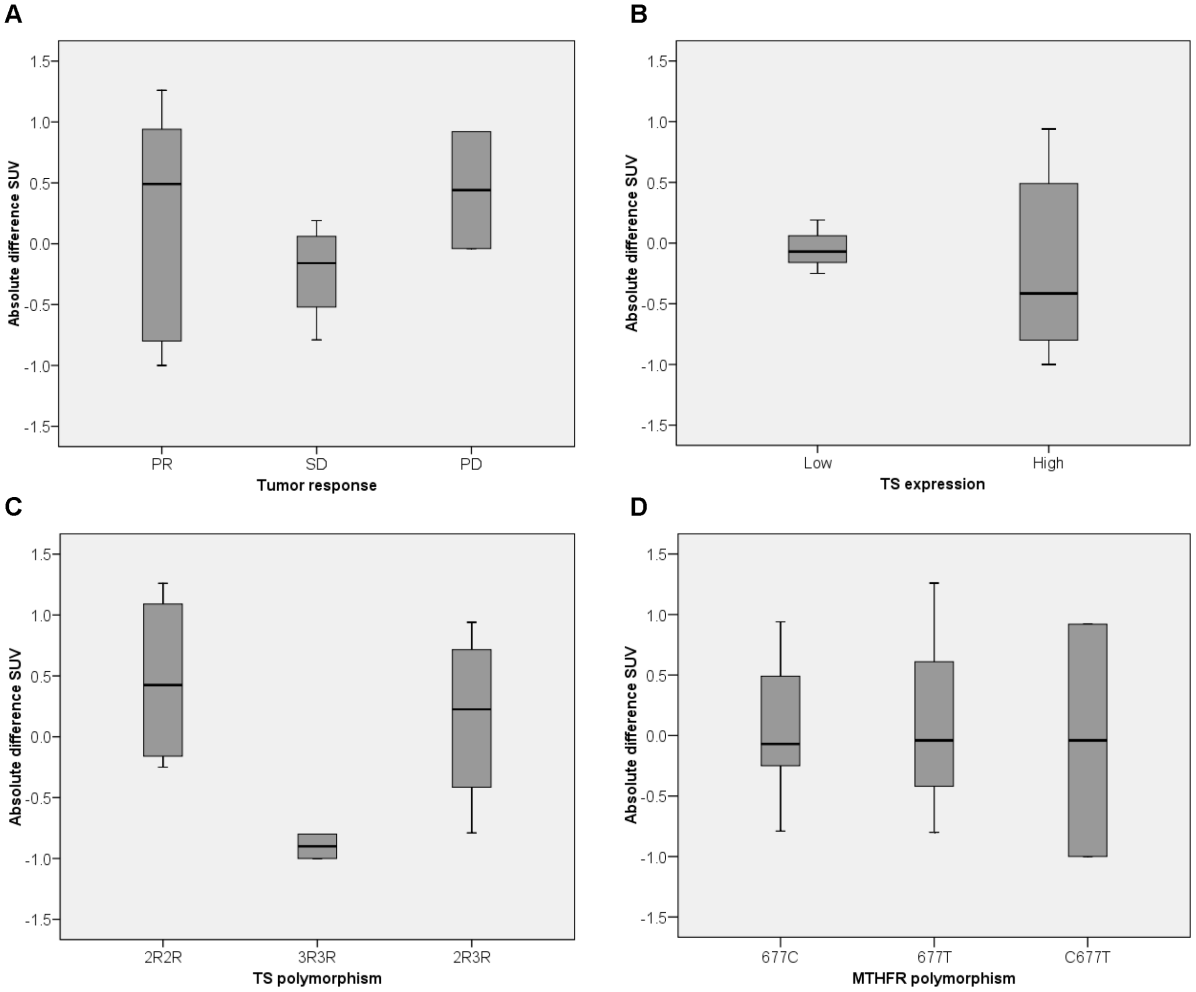
Box plots with absolute differences in SUV. SUV 40–60 min normalised to bodyweight for (A) tumor response after 6 weeks, (B) immunohistochemistry thymidylate synthase expression, (C) thymidylate synthase polymorphism, (D) methylenetetrahydrofolate reductase polymorphism.

After six weeks, response evaluation according to RECIST revealed no patients with complete response (CR), five patients with partial response (PR), seven with stable disease (SD) and two with progressive disease (PD). Baseline SUV was not predictive for tumor response (Kruskal-Wallis test p = 0.86). Tumor response for the eleven PET evaluable patients revealed 5 patients with PR, 4 SD and 2 PD. The mean SUV differences per tumor response group are shown in Figure 6A. ΔSUV was not related with RECIST (Kruskal-Wallis test p = 0.59). In addition, differences in Patlak K_i_, NLR K_i_, k3 or volume of distribution was not related with RECIST (Kruskal-Wallis test p>0.05).

The median TTP was 4.3 months, with a range of 3.0–10.4 months for the complete study population. Baseline SUV from these fourteen patients and TTP were not associated (Cox regression p = 0.54). For the eleven PET evaluable patients the median TTP was 4.2 months, with a range of 3.0–7.4 months. There was no significant association between the difference in SUV and TTP (Log rank, p = 0.96). The median OS was 9.7 months (range 5.1–32.7 months) for the complete group and 13.0 months (range 5.1–30.8 months) for the PET evaluable patients. SUV baseline nor SUV difference were associated with OS (Cox regression, Log rank p = 0.74 and 0.43, respectively).

### Deoxyuridine

**Figure 7:**
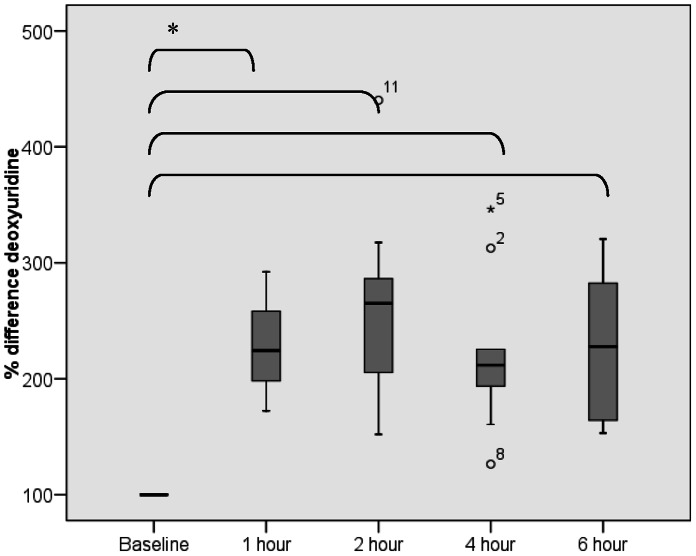
Plasma deoxyuridine over time. *Significant differences tested with paired t-test, p<0.05, indicated by the accolade.

Paired baseline deoxyuridine measurements in plasma showed a consistent value with a mean ± standard deviation of 12.6±7.9 and 11.5±6.0 ng/ml respectively (paired t-test p = 0.995). One hour after administration of pemetrexed deoxyuridine levels significantly rose in all patients (p<0.05) and this persisted until six hours after administration of pemetrexed (see Figure 7). No significant difference in deoxyruridine between one, two, four and six hours after administration of pemetrexed was observed.

### Immunohistochemistry

**Figure 8:**
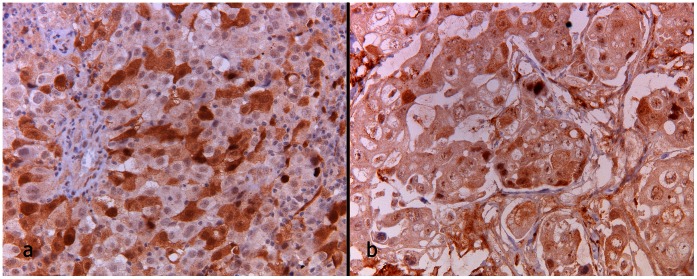
Thymidilate synthase immunohistochemistry (20 ×). In (A) scattered nuclear staining in >50% nuclei, and in (B) around 10% of the nuclei. Note in (A) also concomitant scattered cytoplasmic staining.

IHC for TS was performed in 12 patients. Median H-score was 105 in nucleus and 70 in cytoplasm. Figure 8 illustrates IHC of low and high TS expression. Mean SUV differences for low and high TS expression are shown in Figure 6B (Mann Withney U test p = 0.91).

### TS and MTHFR Polymorphism

TS and MTHFR polymorphisms have been determined for twelve patients. For TS the polymorphisms 2R2R, 2R3R and 3R3R were defined, with respectively five, three and four patients per group. MTHFR polymorphism showed six patients with 677C, three patients with 677T and three patients with C677T. The mean SUV differences per polymorphism are shown in Figure 6C–D. No significant difference in SUV for TS or MTHFR polymorphism was observed (Kruskal-Wallis test p = 0.11 and p = 0.86 respectively). TS or MTHFR polymorphism were not prognostic for tumor response, TTP or OS (Chi square and Kruskal-Wallis p>0.05).

Low or high TS expression measured with IHC correlated with TS polymorphism (Chi-Square p = 0.03). H-score was significantly higher in patients with 2R3R and 3R3R polymorphism compared to 2R2R (Jonckheere-Terpstra p = 0.04). IHC TS expression did not correlate with MTHFR polymorphism (Chi-square p = 0.44).

## Discussion

The present study evaluated the potential of measuring TS-inhibition with ^18^F-FLT PET in NSCLC patients treated with the TS inhibitor pemetrexed. From biologic understanding and preclinical studies, an increase of ^18^F-FLT uptake is expected after pemetrexed administration in responding patients if clinical response is mainly attributed to TS-inhibition. Our data shows that two out of eleven patients had an increased ^18^F-FLT uptake 4 hours after administration of pemetrexed beyond test retest boundaries (15% for SUV) [23], [24]. These two patients had PR measured with RECIST after 6 weeks. However, increased ^18^F-FLT uptake did not correlate with longer TTP or OS.

Two patients showed an unexpected decrease in ^18^F-FLT uptake. The biological explanation for this is unclear. The time-interval of 4 hours should be too short to show the effect of decreased proliferation, although pemetrexed is also known to increase TS function [12], [12,21], lowering its inhibition and hence possibly decrease ^18^F-FLT uptake. The decreased uptake may in addition be explained by reduced perfusion, but we can only speculate this since we did not measure perfusion in this study. K_1_ in these patients were not significantly lowered compared to baseline or to other study patients.

Full kinetic analysis, NLR and Patlak analysis, were evaluated in this study. Correlation between Patlak and SUV was moderate, but did not change between the two scans. Use of NLR, Paltak or volume of distribution did not change our study results compared to SUV.

The full kinetic data showed a reversible two tissue compartment model with 4 rate constants, as determined by Akaike and Schwarz. In the literature an irreversible model for ^18^F-FLT up to 60 minutes has been reported as preferred model [26], [27]. Muzi et al. reported the influence of k_4_ after 90 minutes, implicating a reversible model at that time point [28]. In our study, 7 out of 11 patients had a better fit with a reversible two tissue compartment model. This suggests that k_4_ is different from zero in the majority of the patients in our study population within 60 minutes after injection.

Deoxyuridine plasma levels increased after pemetrexed administration in all patients, which implicates global TS-inhibition. This is in agreement with earlier studies on pemetrexed and other TS inhibitors [29]–[31]. As this increase appeared in all patients, deoxyuridine plasma levels were not able to distinguish which patient had therapeutic TS-inhibition within the tumor. The increase in deoxyuridine plasma levels is most likely due to effective TS-inhibition in normal tissue. This is supported by increased [18F]FLT uptake in bone marrow 4 hours after pemetrexed administration, indicating effective TS inhibition at this time interval. Bone marrow has low TS expression and pemetrexed induced TS-inhibition has been shown to be most effective in tissue with low TS expression [6]. Earlier it was observed in an animal model in bone marrow, that TS was inhibited efficiently by 5FU treatment, but the same dose did not affect liver [32], [33]. Therefore bone marrow is likely to be highly sensitive to TS inhibition by pemetrexed. As a result, hematologic toxicities are also common in patients treated with pemetrexed. Our study confirms early effective TS-inhibition in bone marrow indicated by increased [18F]FLT uptake 4 hours after pemetrexed administration. This non-tumor specific effect of TS-inhibition in bone marrow may serve as a surrogate for other proliferating tissues which clarifies the increased deoxyuridine levels in all patients.

A previous study performed in breast cancer patients (n = 6) revealed an increase in ^18^F-FLT uptake in all but one patient at one hour after treatment with capecitabine [34]. No statistical comparison with clinical outcome was performed. This study in breast cancer patients is the only study with ^18^F-FLT PET scans performed within one day after the start of treatment. The findings in ^18^F-FLT uptake differ from our study results. However, the studies cannot be compared since they evaluate different tumor types, different time intervals and different treatments. In addition, as a 5-FU prodrug, capecitabine has different actions beside TS-inhibition; its metabolic pathway may interfere with thymidine metabolism.

It could be argued that lack of correlation between ^18^F-FLT PET and clinical outcome resulted from inappropriate timing of imaging. The time interval for 4 hours was based on earlier studies with various antifolate TS inhibitors, which showed an optimal increase between 1–4 hr for BGC9331 but between 4–24 hr for another antifolate BGC 945 [*31*]. However, the optimal time point of scanning might not be 4 hours after pemetrexed administration. In addition, acquired drug resistance to therapy could affect the relationship between in principle an effective pharmacodynamic imaging biomarker and the ultimate clinical outcome in time to progression and overall survival.

The response assessment has limitations, as only one patient received pemetrexed monotherapy and the other patients had combination therapy including cisplatin or carboplatin. These differences in therapy affect the clinical outcome. PD after six weeks proofs the lack of therapeutic effect of pemetrexed, but PR could be attributed to the combination therapy with cisplatin or carboplatin. In addition, the clinical outcome can be influenced by lesions outside the field of view of the dynamic ^18^F-FLT PET scan, which is 15.5 cm in the thoracic region.

The limitations of the tracer ^18^F-FLT should be considered. ^18^F-FLT can be in competition with endogenous thymidine. In addition, uptake of ^18^F-FLT is S-phase specific and dependent on the presence of transporter ENT1 and the expression and activity of thymidine kinase 1. Contractor et al. [35] published results of the role of up- and downregulation of the ENT transporter, the most important transporter of ^18^F-FLT into the cell. Soloviev et al. [11] highlighted the limited knowledge of the possible changes in tumors of essential enzymes and transporters involved in the salvage pathway of thymidine after treatment. These still unknown aspects of the biochemical pathway of ^18^F-FLT could affect our study results and should be addressed in future research.

Patients with 2R3R and 3R3R polymorphism showed higher H-scores compared to 2R2R polymorphism, confirming the relation between TS polymorphism and TS expression [36]. TS immuhistochemistry was performed in a standardized way. Unfortunately some limitations apply these measurements. For example, in five patients only metastatic tumor tissue was evaluable because it was technically not feasible to biopsy the primary lung lesion. TS expression in metastasis could differ from the primary. Another limitation is the time-interval between biopsy and ^18^F-FLT PET, this ranged from 1 week to several months. TS expression could change over time. These limitations should be considered interpreting the results from TS expression measured with immuhistochemistry.

TS and MTHFR polymorphism did not correlate with difference in SUV or clinical outcome, but this study was not powered to evaluate this. Therefore, although this pilot study could not confirm it, the correlation between TS and MTHFR polymorphism status and clinical outcome might exist.

### Conclusions

Measuring TS-inhibition in a clinical setting 4 hours after pemetrexed revealed a non-systematic change in tumor ^18^F-FLT uptake, although systemic effects of TS-inhibition were clear. The association with tumor response, time to progression or overall survival was not significant. Further research on the biochemical pathway and uptake patterns of ^18^F-FLT during therapy is needed, including evaluation of the optimal time-interval for scanning.
